# Emergence of Antibiotic-Resistant *Porphyromonas gingivalis* in United States Periodontitis Patients

**DOI:** 10.3390/antibiotics12111584

**Published:** 2023-11-02

**Authors:** Thomas E. Rams, Jacqueline D. Sautter, Arie J. van Winkelhoff

**Affiliations:** 1 Department of Periodontology and Oral Implantology, Temple University School of Dentistry, Philadelphia, PA 19140, USA; jacqueline.sautter@temple.edu; 2 Center for Dentistry and Oral Hygiene, University Medical Center Groningen, University of Groningen, 9713 GZ Groningen, The Netherlands; ajwinkelhoff@laboral.nl

**Keywords:** antibiotics, anti-infective agents, *Porphyromonas gingivalis*, periodontitis, clindamycin, amoxicillin, metronidazole, drug resistance, in vitro, periodontal pocket

## Abstract

Antibiotic resistance patterns of the major human periodontal pathogen *Porphyromonas gingivalis* were assessed over a 20-year period in the United States. Subgingival *P. gingivalis* was cultured pre-treatment from 2193 severe periodontitis patients during three time periods: 1999–2000 (936 patients), 2009–2010 (685 patients), and 2019–2020 (572 patients). The clinical isolates were tested for in vitro resistance to 4 mg/L for clindamycin and doxycycline, 8 mg/L for amoxicillin, and 16 mg/L for metronidazole, with a post hoc combination of data for metronidazole plus amoxicillin. Clindamycin-resistant *P. gingivalis* was significantly more prevalent in 2009–2010 (9.1% of patients) and 2019–2020 (9.3%; 15-fold increase) as compared to 1999–2000 (0.6%). *P. gingivalis* resistance to amoxicillin also significantly increased from 0.1% of patients in 1999–2000 to 1.3% in 2009–2010 and 2.8% (28-fold increase) in 2019–2020. *P. gingivalis* resistance to metronidazole, metronidazole plus amoxicillin, and doxycycline was low (≤0.5% prevalence), and statistically unchanged, over the 20-year period. These findings are the first to reveal marked increases over 20 years in clindamycin-resistant and amoxicillin-resistant *P. gingivalis* in United States periodontitis patients. Increased antibiotic resistance of *P. gingivalis* and other periodontitis-associated bacteria threatens the efficacy of periodontal antimicrobial chemotherapy.

## 1. Introduction

The World Health Organization and the United Nations have declared the rapid rise in antibiotic resistance in pathogenic bacteria as a global crisis threatening control of bacterial infections [1]. The United States Centers for Disease Control and Prevention estimates that at least 35,000 people annually die of antibiotic-resistant infections in the United States [2]. Surveillance surveys to monitor the extent of antibiotic resistance in pathogenic bacteria are considered essential for evaluating the efficacy of antibiotic stewardship programs [3,4]. However, data on antimicrobial resistance among periodontal bacterial pathogens in the human oral cavity are presently limited and largely cover only single time points [5,6]. 

*Porphyromonas gingivalis* is a major periodontal disease bacterial pathogen [7]. This Gram-negative anaerobic rod is able to enter gingival tissues after colonizing subgingival tooth biofilms [8], express pro-inflammatory and immune-impairing virulence factors [9,10,11,12], and act as a “keystone” pathogen facilitating dysbiosis in subgingival microbial communities [13]. *P*. *gingivalis* is strongly associated with severe periodontitis [14] and peri-implantitis lesions [15] and may play a role in the etiology of oral, oropharyngeal, and esophageal cancer [16]. Extraoral dissemination of *P. gingivalis* has been linked to adverse disruption of the gut microbiome [17], development of acute infections at various body sites [18], and the etiopathogenesis of atherosclerosis, rheumatoid arthritis, diabetes mellitus, respiratory diseases, and Alzheimer’s disease [19,20,21,22,23,24,25,26].

Conventional non-surgical and surgical periodontal therapy may fail to adequately remove *P. gingivalis* from deep periodontal pockets, predisposing patients to impaired treatment outcomes [27,28,29,30,31], further periodontal breakdown [32,33,34,35], and an increased risk of *P. gingivalis*-influenced systemic diseases [19,20,21,22,23,24,25,26]. As a result, systemic antibiotics are often prescribed to refractory periodontitis patients with *P. gingivalis* persisting in their post-treatment subgingival microbiota [31,36,37,38,39,40,41,42,43,44]. Metronidazole, amoxicillin alone or in combination with metronidazole, clindamycin, and doxycycline are among the oral antibiotics frequently recommended as adjuncts to conventional mechanical/surgical treatment of periodontitis [31,36,37,38,39,40,41,42,43,44,45]. 

Little is known about the present-day antibiotic susceptibility of periodontal *P. gingivalis* in the United States [46,47]. *Porphyromonas gingivalis* clinical isolates in the United States prior to 2010 were rarely antibiotic-resistant [46,48], with only ≤0.6% of 312 species-positive patients yielding *P. gingivalis* resistant to clindamycin, amoxicillin, metronidazole, metronidazole plus amoxicillin, or doxycycline [46]. 

Because of a lack of longitudinal surveillance data, it is not known if the antibiotic resistance profile of subgingival *P. gingivalis* in the United States has changed similarly to recent increases in the antibiotic resistance of other anaerobic bacteria at non-oral infection sites [49]. To address this issue, this study examined temporal changes in the antibiotic resistance patterns of subgingival *P. gingivalis* in United States periodontitis patients over a 20-year period.

## 2. Materials and Methods

### 2.1. Patients

The study patients were selected for three time periods over 20 years (1999–2000, 2009–2010, and 2019–2020) from a retrospective search of consecutive de-identified laboratory records at the Oral Microbiology Testing Service (OMTS) Laboratory at Temple University School of Dentistry, Philadelphia, Pennsylvania. Patients were identified from the record search and included in the present study, forming 3 patient groups as their pre-treatment subgingival biofilms were consecutively evaluated by the OMTS Laboratory. The patients were all adults aged ≥ 35 years old, culture-positive for subgingival *P. gingivalis*, and diagnosed with severe periodontitis (equivalent to at least stage III periodontitis) [50] by periodontists in private dental practices in the United States. A total of 936 patients were evaluated for antibiotic-resistant *P. gingivalis* in 1999–2000, 685 in 2009–2010, and 572 in 2019–2020, resulting in an overall total of 2193 study patients. 

Each patient clinically exhibited interproximal probing depths > 6 mm with bleeding on probing on ≥3 teeth, which strongly correlates (94.1% positive predictive value) with severe periodontal attachment loss in adult patients [51]. Available patient data were inadequate for determining the extent and grade of periodontitis in the study patients and for differentiating between stage III and stage IV periodontitis cases. Persons with molar-incisal pattern (aggressive) periodontitis or with a history of antibiotic use within the previous 6 months were excluded. Most of the study patients originated geographically from periodontal specialty practices in the mid-Atlantic region of the United States (Maryland, Pennsylvania, New Jersey, Delaware, New York, Virginia, West Virginia, and the District of Columbia). 

The OMTS Laboratory was licensed during the 20-year study time period for high-complexity bacteriologic analysis and bacterial susceptibility testing by the Pennsylvania Department of Health and was federally certified by the United States Centers for Medicare and Medicaid Services to be in compliance with Clinical Laboratory Improvement Amendments (CLIA)-mandated proficiency testing, quality control, patient test management, personnel requirements, and quality assurance standards required of clinical laboratories engaged in diagnostic testing of human specimens in the United States [52]. All laboratory procedures throughout the study period were performed on a standardized basis by personnel who were masked to the clinical status of the study patients and their inclusion in the present study. A single laboratory director licensed by the Pennsylvania Department of Health (author T.E.R.) supervised and reviewed all microbiological testing over the 20-year period. Two experienced laboratory technicians (including the author J.D.S.), calibrated with each other and the laboratory director, processed all of the study patient subgingival specimens and cultures. 

This study was approved by the Temple University Human Subjects Institutional Review Board and was conducted in accordance with the Helsinki Declaration of 1975, as revised in 2013. The Temple University Human Subjects Institutional Review Board reviewed the study protocol and judged it to be exempt from further ethical approval, since the retrospective analysis of de-identified laboratory data did not involve any patient contact, interaction, or intervention.

### 2.2. P. gingivalis Clinical Isolates

*P. gingivalis* was isolated from pre-treatment subgingival biofilm samples from each study patient. The subgingival specimens were obtained by the patient’s diagnosing periodontists, following a standardized subgingival sampling protocol previously described [46]. After isolation with cotton rolls and removal of saliva and supragingival plaque, 1 to 2 sterile, absorbent paper points (Johnson & Johnson, East Windsor, NJ, USA) were advanced into 3 to 5 deep (>6 mm) periodontitis sites with bleeding on probing for 10 s. After removal, all paper points per patient were pooled into a screw-topped glass vial containing the anaerobically prepared and stored viability medium Gothenburg anaerobic (VMGA) III transport medium [53]. After transport within 24 h to the OMTS Laboratory, the specimen vials were processed as previously described [46]. In brief, the vials were first warmed to 35 °C to liquefy the VMGA III transport medium and vortexed at the maximal mixer setting for 45 s to mechanically disperse bacterial cells from the paper points. Serial 10-fold dilutions of the bacterial suspensions were prepared in Möller’s VMG I anaerobic dispersion solution comprising pre-reduced, anaerobically sterilized 0.25% tryptose, 0.25% thiotone E peptone, and 0.5% NaCl [54]. Then, 0.1 mL dilution aliquots were spread with a sterile bent-glass rod onto nonselective enriched Brucella blood agar (EBBA) primary isolation plates [46] comprising 4.3% Brucella agar supplemented with 0.3% bacto-agar, 5% defibrinated sheep blood, 0.2% hemolyzed sheep red blood cells, 0.0005% hemin, and 0.00005% menadione. The plates were incubated at 37 °C for 7 days in an anaerobic atmosphere containing 85% N_2_, 10% H_2_, and 5% CO_2_. 

*P. gingivalis* was identified on EBBA primary isolation plates as circular, dome-shaped, dark-pigmented (brown to tan), raised surface colonies that lacked brick-red autofluorescence under long-wave ultraviolet light (365 nm wavelength) [55] but exhibited a positive CAAM test outcome for trypsin-like enzyme activity [56]. A subset of these phenotypically identified isolates from 38 patients were subjected to matrix-assisted laser desorption/ionization time-of-flight (MALDI-TOF) mass spectrometry, as previously described [57], with 100% definitively confirmed to be *P. gingivalis*. The proportional recovery of *P. gingivalis* from each patient was calculated as the percentage of *P. gingivalis* colony-forming units relative to total subgingival anaerobic viable counts, as determined on nonselective EBBA primary isolation plates. The detection threshold for *P. gingivalis* on EBBA is estimated to be 1 colony per 500–1000 colonies (0.1–0.2% of total viable counts) [58].

### 2.3. P. gingivalis In Vitro Antibiotic Resistance Testing

In vitro *P. gingivalis* antibiotic resistance testing was performed using a direct plating method as previously described [46], which correlates well (*r*^2^ = 0.99) with the Clinical and Laboratory Standards Institute (CLSI)-approved agar dilution susceptibility assay for identifying antibiotic-resistant periodontal microorganisms [59]. 

In brief, 0.1 mL aliquots of subgingival biofilm dilutions for each patient were inoculated onto EBBA primary isolation plates supplemented with either amoxicillin at 8 mg/L, clindamycin at 4 mg/L, doxycycline at 4 mg/L, or metronidazole at 16 mg/L (all antimicrobials were obtained as pure powder from Sigma-Aldrich, St. Louis, MO, USA), which represent non-susceptible or resistant breakpoint drug concentrations for amoxicillin, clindamycin, and metronidazole against anaerobic bacteria as recommended by the CLSI [60], and for doxycycline as recommended by the French Society for Microbiology [61]. These antibiotics are frequently recommended for adjunctive oral chemotherapy in the treatment of human periodontitis [31,36,37,38,39,40,41,42,43,44,45] and were employed for in vitro antibiotic resistance testing of *P. gingivalis* and other putative periodontal bacterial pathogens in the OMTS Laboratory during the 20-year study period. After anaerobic incubation for 7 days, *P. gingivalis* isolates growing on antibiotic-supplemented media were considered resistant to the incorporated antibiotic concentration, as previously described [46]. A subset of *P. gingivalis* isolates growing on antibiotic-supplemented media were subjected to MALDI-TOF mass spectrometry identification testing [57] as part of the OMTS Laboratory’s quality control CLIA certification requirements and confirmed to be *P. gingivalis*. *Bacteroides thetaiotaomicron* ATCC 29741, *Clostridium perfringens* ATCC 13124, and a multi-antibiotic-resistant clinical periodontal isolate of *Fusobacterium nucleatum* were used as positive and negative quality controls for all antibiotic resistance testing on drug-supplemented EBBA plates. 

In a separate pilot study, 6 *P. gingivalis* clinical isolates exhibiting in vitro resistance to 4 mg/L clindamycin with the direct plating method [46] were subjected to in vitro clindamycin gradient strip susceptibility testing [62]. Pure cell suspensions of each *P. gingivalis* strain were first prepared and adjusted to a 0.5 McFarland turbidity standard using sterile VMG I anaerobic dispersion solution and then streaked with sterile cotton-tipped swabs onto EBBA plates. After drying, predefined antibiotic gradient strips (E-test, bioMérieux, Durham, NC, USA) containing clindamycin were applied onto the inoculated media surfaces. After 48 h of anaerobic incubation at 37 °C, the intersection between the border of *P. gingivalis* growth and the antibiotic gradient strip drug scale was read to determine in vitro minimum inhibitory concentration (MIC) values, following the manufacturer’s instructions. Clindamycin-resistant strains of *P. gingivalis* were identified using CLSI interpretative guidelines for clindamycin against anaerobic bacteria [60]. *B. thetaiotaomicron* ATCC 29741 was used as a quality control strain in clindamycin gradient strip susceptibility testing.

### 2.4. Data Analysis

Descriptive analysis characterized the study patients and tabulated per patient the proportional cultivable recovery of *P. gingivalis* and the prevalence and subgingival proportions of antibiotic-resistant *P. gingivalis*. Means and standard deviation (SD) were calculated for continuous variables, and frequencies and percentages for categorical variables. Antibiotic resistance data were combined and analyzed post hoc from amoxicillin- and metronidazole-supplemented EBBA culture plates. This was based on previous studies demonstrating excellent agreement (98.5%) between periodontal pathogen antibiotic resistance patterns as determined from EBBA plates jointly supplemented with both amoxicillin and metronidazole, as compared to a post hoc combination of findings from EBBA plates individually supplemented with amoxicillin or metronidazole [63]. One-way analysis of variance (ANOVA), followed by Tukey’s HSD test, compared differences in mean patient age and mean percent of recovered subgingival *P. gingivalis* between the patient groups. Fisher’s exact test examined differences between the patient groups in the proportion of males, and the percentage of patients with antibiotic-resistant *P. gingivalis* in 2009–2010 and 2019–2020, as compared to 1999–2000, as well as between 2009 and 2010 and 2019 and 2020, for each of the tested antibiotics. A *p*-value ≤ 0.05 was required for statistical significance. The PC-based STATA/SE 16.1 for Windows (StataCorp PL, College Station, TX, USA) 64-bit statistical software package was used in the data analysis.

## 3. Results

### 3.1. Quality Control

Test results were within expected ranges and outcomes for the three quality control bacterial strains subjected to in vitro amoxicillin, clindamycin, doxycycline, and metronidazole breakpoint resistance testing, as well as the quality control strain evaluated with clindamycin gradient strips.

### 3.2. Patients and Subgingival P. gingivalis Recovery

Table 1 provides selected features of the three patient groups with severe periodontitis.

No statistically significant gender differences were found between the three patient groups (*p*-values > 0.05). The mean age of 1999–2000 patients was slightly but significantly lower, and the mean percentage of subgingival *P. gingivalis* in 2019–2020 patients was slightly but significantly lower than in the other two patient groups (*p*-values < 0.05). 

### 3.3. P. gingivalis In Vitro Antibiotic Resistance Testing

**Figure 1:**
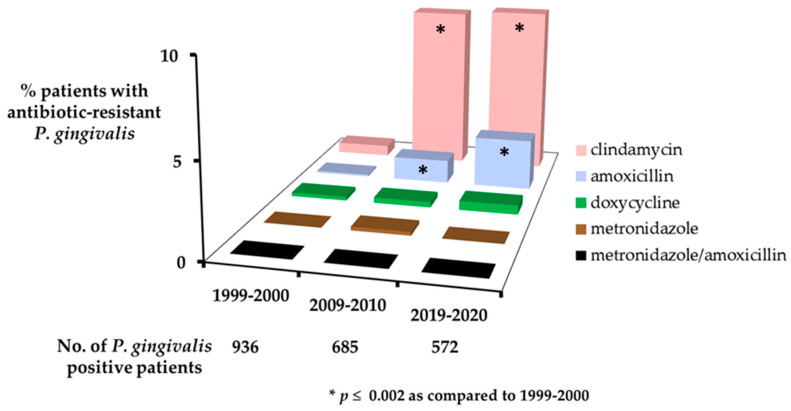
Prevalence of antibiotic-resistant *P. gingivalis* in United States periodontitis patients over 20 years.

Table 2 and Figure 1 display the distribution by study time period of patients yielding *P. gingivalis* resistant in vitro to non-susceptible/resistant breakpoint concentrations of the test antibiotics. 

In vitro resistance of *P. gingivalis* to 4 mg/L clindamycin was rare in 1999–2000, with resistant isolates found in only 0.6% of 936 *P. gingivalis* culture-positive patients. However, the prevalence of clindamycin-resistant *P. gingivalis* was significantly greater in 2009–2010, with a 15-fold increase in species resistance to 9.1% of the patients, and in 2019–2020, with a 15.5-fold increase in species resistance to 9.3% of the patients, as compared to the levels found in 1999–2000 (Table 2). Differences in the prevalence of clindamycin-resistant *P. gingivalis* were statistically significant between patients evaluated in 1999–2000 versus those sampled at later time periods (*p* < 0.001) but were not significantly different between patients evaluated in 2009–2010 and 2019–2020 (*p* = 0.922).

In vitro resistance of *P. gingivalis* to 8 mg/L amoxicillin was also rare in 1999–2000, with resistant isolates found in only one (0.1%) patient (Table 2). A significantly higher prevalence of amoxicillin-resistant *P. gingivalis* was found among patients evaluated in 2009–2010 (1.3%; a 13-fold increase) and 2019–2020 (2.8%; a 28-fold increase) as compared to 1999–2000 (*p* ≤ 0.002), but not between patients in 2009–2010 and 2019–2020 (*p* = 0.069). 

No or negligible (≤0.3% of patients) in vitro *P. gingivalis* resistance, and no statistically significant temporal changes in drug resistance patterns, were found with metronidazole at 16 mg/L, the joint effects of metronidazole at 16 mg/L plus amoxicillin at 8 mg/L, or doxycycline at 4 mg/L (Table 2). Only 1 of the total 2193 *P. gingivalis* culture-positive study patients yielded *P. gingivalis* resistant in vitro to 16 mg/L metronidazole, and none had *P. gingivalis* resistant to both metronidazole at 16 mg/L and amoxicillin at 8 mg/L. 

All *P. gingivalis* clinical isolates identified as resistant in vitro to 4 mg/L clindamycin with the direct plating method and then evaluated with gradient strip susceptibility testing were confirmed to be resistant to clindamycin, with all MIC values > 4 mg/L.

## 4. Discussion 

With a global rise in antibiotic resistance documented for many bacterial species in the human microbiome [49,64], it is relevant to evaluate whether similar changes occur in the subgingival microbiota of periodontitis patients. In this study of United States periodontitis patients, from the negligible levels initially detected in 1999–2000, the prevalence of subgingival *P. gingivalis* resistant to breakpoint concentrations of clindamycin significantly increased by 15-fold (to 9.3% of patients) and resistant to amoxicillin by 28-fold (to 2.8% of patients) over a 20-year period. In comparison, no significant increases over 20 years were found with the initially low baseline levels of *P. gingivalis* resistance to metronidazole, metronidazole plus amoxicillin, and doxycycline. These findings are the first United States-specific data documenting 20-year temporal changes in the prevalence of antibiotic-resistant *P. gingivalis* in subgingival biofilms of periodontitis patients. 

Importantly, the prevalence of antibiotic-resistant *P. gingivalis* and other periodontal pathogens varies considerably between countries and geographic regions [5,6], which underscores the need for region-specific surveillance monitoring of antibiotic resistance trends among periodontopathic bacterial species. Pioneering studies by van Winkelhoff et al. [65,66] found differences in antibiotic resistance patterns of periodontal pathogens between the Netherlands and Spain to be correlated with differences in antibiotic exposure between the two countries. Emergence of antibiotic resistance in the subgingival microbiota in specific population groups is likely multifactorial and driven by several selective pressures, including excessive antibiotic prescription/consumption practices, incorrect use of antibiotics, environmental exposure to antibiotic-laden livestock, farmed fish, agricultural waste, and municipal wastewater [64], and more recently, the use of antidepressant medications [67]. Since a wide array of antibiotic resistance genes are frequently present as an oral resistome in the human oral cavity [68,69], the marked increases in clindamycin-resistant and amoxicillin-resistant *P. gingivalis* over time in the present study may stem from horizontal transfer and phenotypic expression of resistance genes for these antibiotics from other oral microorganisms [48,70], spurred on by high exposure to antibiotics and antidepressant medications in the United States population [64,67].

The increase in clindamycin-resistant *P. gingivalis* to a 9.3% prevalence level in the United States is alarming and exceeded worldwide only by a 23.5% frequency of clindamycin-resistant *P. gingivalis* strains in Colombia in South America, where there is greater population exposure to antibiotic-containing over-the-counter products than in the United States [71,72]. Meanwhile, clindamycin resistance in *P. gingivalis* is reported to be absent or low in the Netherlands, Germany, Switzerland, Japan, Spain, Italy, Turkey, Iran, and Brazil [5,6,73]. Consistent with our findings in the United States, anaerobic bacteria residing in non-oral body sites have also recently exhibited large increases in clindamycin resistance linked to the spread of rRNA-methylase-encoding *erm* antibiotic resistance genes [74]. 

These findings have important clinical implications for United States periodontitis patients. Clinical treatment failures in periodontics have occurred when antibiotic therapy was given to patients where the targeted periodontal pathogens were resistant to the selected medication [75]. Similarly, poorer clinical and bacteriologic responses to antibiotic therapies are found in medical infections when antibiotic-resistant pathogens are present [76,77]. Clindamycin was shown over 3 decades ago to be useful in resolving *P. gingivalis*-associated refractory cases of periodontitis and arresting progressive periodontal attachment loss [78]. At the time, clindamycin was found to be highly active against subgingival *P. gingivalis* clinical isolates [79], supporting recommendations of clindamycin as a therapeutic option in the treatment of periodontitis responding poorly to conventional mechanical/surgical treatment regimens [36,37,38,42,45]. Clindamycin is additionally recommended by the American Academy of Periodontology as an empirical antibiotic choice in the treatment of periodontal abscesses [37], where *P. gingivalis* is often part of the associated microbial etiology [80]. However, due to the increased prevalence of clindamycin-resistant *P. gingivalis*, as documented in the present study, these recommendations likely need to be re-considered and appropriately modified. In our opinion, empirical use of clindamycin in the treatment of *P. gingivalis*-associated periodontitis and periodontal abscesses in the United States should be minimized in the absence of *P. gingivalis* antibiotic susceptibility testing to limit further emergence of clindamycin-resistant *P. gingivalis* strains in the population. 

*P. gingivalis* in vitro resistance to amoxicillin increased to 2.8% of isolates in the United States over the 20 years under study. This compares to a much higher resistance rate (25%) to amoxicillin among *P. gingivalis* clinical isolates in Columbia [71] and slightly exceeds the widespread absence of *P. gingivalis* resistance to amoxicillin elsewhere in the world [5,6,73]. 

The low-to-negligible levels of *P. gingivalis* resistance to metronidazole and metronidazole plus amoxicillin in United States patients is consistent with global data [5,6,73], except for a high *P. gingivalis* metronidazole resistance rate (≥21.6%) detected in Columbia [71,72]. Only 1 study patient yielded *P. gingivalis* resistant in vitro to metronidazole, and none had *P. gingivalis* resistant to both metronidazole and amoxicillin, out of the 2193 *P. gingivalis*-positive periodontitis patients studied. The low-to-negligible level of metronidazole resistance among *P. gingivalis* and other anaerobic periodontal pathogens in United States periodontitis patients [46,47,81] supports the continued use of metronidazole and the combination of metronidazole plus amoxicillin as possible adjuncts to conventional mechanical-based periodontal therapy for appropriately selected patients in the United States [31,36,37,38,39,40,41,42,43,44].

Doxycycline was found to remain almost uniformly active in vitro against subgingival *P. gingivalis* in the United States (Table 2), similar to elsewhere in the world [5,6,73]. However, the poor in vivo gastrointestinal absorption of orally administered doxycycline in approximately 50% of periodontitis patients, resulting in negligible-to-minimal gingival crevicular fluid drug levels [82], markedly limits the potential clinical use and therapeutic value of systemic doxycycline in periodontal practice. 

The increased antibiotic resistance of *P. gingivalis* in the present study parallels similar increases in the antibiotic resistance of *Parvimonas micra* [81], another major bacterial pathogen in human periodontitis [7]. Over a 10-year period between 2006 and 2016, the prevalence of subgingival *P. micra* resistance to clindamycin increased by 23.7-fold (to 47.3% of patients) and that resistant to doxycycline by 37.7-fold (to 11.3% of patients) in United States periodontitis patients [81]. There is an urgent need to evaluate additional putative periodontal bacterial pathogens in United States periodontitis patients for potential changes in their antibiotic susceptibility patterns.

Strengths of the present study include the relatively large number of severe periodontitis patients evaluated and the fairly long evaluation period of 20 years. Reliable identification of cultivable *P. gingivalis* was achieved using standardized and validated phenotypic criteria documented to have 100% concordance with definitive identification of *P. gingivalis* via MALDI-TOF mass spectrometry [57], which in turn has 100% concordance with identification of *P. gingivalis* via molecular 16S rRNA gene sequencing analysis [83]. The standardized antibiotic resistance testing protocol, validated to have near-perfect agreement with the CLSI-approved agar dilution antibiotic susceptibility assay [59], was employed during the 20-year study time period by a single laboratory director and two experienced laboratory technicians in a CLIA-certified clinical microbiology laboratory licensed by a state health department for high-complexity bacteriologic analysis and bacterial susceptibility testing of subgingival biofilms. This increases confidence in the reliability of the study data and minimizes the possibility that the detected changes in the antibiotic susceptibility of *P. gingivalis* were merely due to laboratory error, changes in laboratory personnel, or examiner drift over time. 

The present study data have a number of limitations. Different patients were evaluated at each of the study time periods, with no longitudinal cohort of patients evaluated. The patient groups differed, with one group having a slightly younger average patient age and another with <3% lower mean proportional levels of subgingival *P. gingivalis* than the other two groups (Table 1). However, since *P. gingivalis* in all patient groups averaged >10% of the cultivable subgingival microbiota, it is unlikely that these relatively small group differences impacted the present study results. More detailed demographic information for the study patients, such as their history of antibiotic and antidepressant drug use, was not available. The patients were not necessarily statistically representative of periodontitis patients throughout the United States. No clinical or radiographic evaluations of the study patients were made by calibrated examiners separate from the diagnostic information submitted by the participating private practice periodontists. MIC values of the test antibiotics against *P. gingivalis* were not determined, and carriage of antibiotic resistance genes by the *P. gingivalis* clinical isolates was not studied. It is important to note carriage of antibiotic resistance genes does not necessarily lead to phenotypic expression of bacterial antibiotic resistance since such genes in periodontal bacterial pathogens often remain silent and unexpressed [84]. Additional mechanistic studies are needed to determine the basis for the increased levels of *P. gingivalis* resistance to clindamycin and amoxicillin in United States periodontitis patients. 

Nevertheless, the present microbiological surveillance data better inform dental professionals in the United States about antibiotic resistance trends in the major human periodontal pathogen *P. gingivalis* and may serve as part of a new antibiotic resistance baseline for dental antibiotic stewardship programs in the United States [85]. Additional population-specific surveillance monitoring is needed in the United States and other countries to further track the development of antibiotic resistance among periodontitis-associated bacterial species.

## 5. Conclusions

Systematic surveillance of the antibiotic susceptibility of target bacterial pathogens in periodontitis is necessary to detect changes in antibiotic resistance patterns, particularly when systemic/local antibiotic therapy is employed in clinical periodontal practice. The present study found clindamycin-resistant and amoxicillin-resistant *P. gingivalis* markedly increased in prevalence over a 20-year period in United States adults with severe periodontitis, whereas no significant temporal changes were found with low levels of *P. gingivalis* resistance to metronidazole, metronidazole plus amoxicillin, and doxycycline. These findings are the first to document the emergence of antibiotic-resistant periodontal *P. gingivalis* in the United States. They indicate a need for clinical caution when employing clindamycin or amoxicillin by itself in the treatment of United States periodontitis patients. Increased antibiotic resistance in *P. gingivalis* and other periodontitis-associated bacteria threatens the efficacy of periodontal antimicrobial chemotherapy. 

## Figures and Tables

**Table 1:** Features of 2193 *P. gingivalis*-positive study patients with severe periodontitis.

Feature	Patient Group		
Time period	1999–2000	2009–2010	2019–2020
No. of patients	936	685	572
% male	49.7	48.6	47.6
Mean age, years ± SD	53.1 ± 10.6 *	55.9 ± 11.2	55.7 ± 12.7
Age range, years	35–81	35–86	35–87
Mean% *P. gingivalis* ± SD	13.3 ± 15.3	12.9 ± 12.6	10.6 ± 12.1 *
Range% *P. gingivalis*	0.1–78.9	0.1–68.9	0.1–69.3

* significantly different than other patient groups, *p*-values < 0.05.

**Table 2:** Distribution of United States periodontitis patients with antibiotic-resistant *P. gingivalis*.

	Patient Group		
Antibiotic	1999–2000 (N = 936)	2009–2010 (N = 685)	2019–2020 (N = 572)
No. (%) patients with clindamycin-resistant *P. gingivalis*	6 (0.6)	62 (9.1) *	53 (9.3) *
Mean % drug-resistant *P. gingivalis* ± SD	19.2 ± 16.8 ^‡^	7.2 ± 9.2	11.3 ± 15.4
No. (%) patients with amoxicillin-resistant *P. gingivalis*	1 (0.1)	9 (1.3) *	16 (2.8) *
Mean % drug-resistant *P. gingivalis* ± SD	1.7	9.3 ± 11.5	10.0 ± 17.2
No. (%) patients with doxycycline-resistant *P. gingivalis*	2 (0.2)	2 (0.3)	3 (0.5)
Mean % drug-resistant *P. gingivalis* ± SD	14.5 ± 6.4	7.7 ± 3.4	9.7 ± 6.4
No. (%) patients with metronidazole-resistant *P. gingivalis*	0	1 (0.2)	0
Mean % drug-resistant *P. gingivalis* ± SD	0	1.6	0
No. (%) patients with metronidazole/amoxicillin-resistant *P. gingivalis*	0	0	0
Mean drug-resistant % *P. gingivalis* ± SD	0	0	0

^‡^ Levels of subgingival *P. gingivalis* in patients with test antibiotic-resistant strains, * % of affected patients significantly different than 1999–2000 patient group, *p*-values ≤ 0.002.

## Data Availability

Not applicable.
